# The unhealthy food environment does not modify the association between obesity and participation in the Supplemental Nutrition Assistance Program (SNAP) in Los Angeles County

**DOI:** 10.1186/s12889-016-4011-z

**Published:** 2017-01-14

**Authors:** M. Pia Chaparro, Gail G. Harrison, May C. Wang, Edmund Y. W. Seto, Anne R. Pebley

**Affiliations:** 1 Institute for Social and Economic Research (ISER), University of Essex, Wivenhoe Park, Colchester, Essex CO4 3SQ UK; 2 Department of Community Health Sciences, Fielding School of Public Health, University of California Los Angeles (UCLA), Los Angeles, CA 90095 USA; 3 Department of Environmental & Occupational Health Sciences, School of Public Health, University of Washington, Office: HSB F-226C, Box: 357234, Seattle, WA 98195 USA; 4 Department of Sociology, University of California Los Angeles (UCLA), Los Angeles, CA 90095 USA

**Keywords:** SNAP, Obesity, Food environment, Los Angeles County, Effect modification, Multilevel analysis

## Abstract

**Background:**

Participation in the Supplemental Nutrition Assistance Program (SNAP) has been linked to an increased risk of obesity, but not much is known about the mechanisms behind this association. The objective of this study was to determine if the neighborhood density of unhealthy food outlets modifies the association between obesity and participation in SNAP.

**Methods:**

Data comes from the first wave of the Los Angeles Family and Neighborhood Survey; included are a subsample of adults (18+ years) who were SNAP participants or eligible non-participants (*N* = 1,176). We carried out multilevel analyses with obesity (BMI ≥ 30 Kg/m^2^), SNAP participation, and the neighborhood density of unhealthy food outlets as dependent, independent and modifying variables, respectively, controlling for age, gender, race/ethnicity, marital status, working status, mental health, and neighborhood poverty.

**Results:**

SNAP participants had double the odds of obesity compared to eligible non-participants (OR = 2.02; 95%CI = 1.44-2.83). However, the neighborhood density of unhealthy food outlets did not modify this association.

**Conclusions:**

SNAP participation was associated with higher odds of obesity in our primarily Hispanic sample in Los Angeles County, with no effect modification found for the unhealthy portion of the food environment. More research is needed with additional food environment measures to confirm our null findings. Additional research is needed to elucidate the mechanisms linking SNAP participation and obesity as they remain unclear.

## Background

The Supplemental Nutrition Assistance Program (SNAP) is the largest federal food assistance program in the U.S., serving as a safety net against food insecurity for low-income families and reaching approximately 48 million people monthly [1]. Research has shown that SNAP participation is associated with obesity, particularly among women [2, 3]. Though controversy exists in terms of the causality of this association [4], the high volume of people reached by SNAP, makes it an important target for research investigating ways of curbing the obesity epidemic [5].

In their 2012 review, DeBono et al. [3] called for more place-based research on the association between obesity and SNAP participation. Specifically, the authors highlight the need to understand *how* the neighborhood accessibility (and quality) of SNAP stores would affect SNAP participants’ weight status [3]. In recent years, the food environment has been studied in reference to both obesity [6] and SNAP [7, 8], with some studies focused on the role of the food environment (particularly the healthy component) in the SNAP participation – obesity relationship [9]. Moreover, a recent evaluation on the adequacy of SNAP allotments concluded that aspects of the nutritional environment need to be taken into account when evaluating and revising such allotments [10], highlighting the importance of the food environment for SNAP participants.

The objective of this study was to investigate if the neighborhood density of unhealthy food outlets is an effect modifier in the association between SNAP participation and obesity among adults in Los Angeles County. Los Angeles is the largest county in the U.S. with approximately 10 million inhabitants, 3.5 million of which are foreign-born residents. It contains segregated as well as demographically diverse communities [11], which makes it an interesting location to examine this research question. The prevalence of obesity among adults in Los Angeles was 25% in 2011–2012 [12], with significant racial/ethnic and geographical disparities [13]. We hypothesized that a higher neighborhood density of unhealthy food outlets would increase the magnitude of the association between SNAP participation and obesity.

## Methods

We used data from the Los Angeles Family and Neighborhood Survey (L.A.FANS, 1^st^ wave, 2000–2002), which is based on a representative sample of 65 census tracts in Los Angeles County randomly selected from three strata: non-poor (bottom 60% of the poverty distribution) poor (60–89% of the poverty distribution), and very poor (top 10% of the poverty distribution). Within each census tract, a sample of 50 households obtained through a multi-stage stratified sampling design was interviewed. Further details on the sample and data collection can be found elsewhere [14]. We focused on a sub-sample of adults (age ≥ 18 years; *n* = 1176) who were either SNAP participants or eligible non-participants. Eligible non-participants were identified based on income (gross income and net income <130 and <100% of the federal poverty level, respectively), asset ownership (<$2000 or < $3000 if a senior lived in the household), and immigration status (US citizens or permanent residents), following California SNAP eligibility rules [15], as explained in detail elsewhere [16].

Our dependent variable was obesity (Body Mass Index (BMI) ≥ 30 Kg/m^2^), with BMI estimated from self-reported weights and heights, and our main independent variable was SNAP participation (yes/no).

We used the National Establishment Time-Series (NETS) database, based on Dun and Bradstreet archival establishment data, to extract information on the neighborhood food environment of L.A.FANS respondents. Using Standard Industry Classification (SIC) codes and the years in which the businesses were active, we obtained counts per census tract for fast food restaurants (chain and independent, including pizza restaurants), chain convenience stores, and liquor stores (which in Los Angeles sell mostly convenience food in addition to alcohol). We summed these counts to create a count of unhealthy food outlets and then estimated the density of unhealthy food outlets (count per square mile) for each census tract where L.A.FANS respondents lived in the years in which the survey took place (2000–2002). Although unhealthy foods may be sold in other food outlets (e.g. grocery stores and supermarkets), we decided to include only the above outlets which sell mostly energy-dense, high-sugar and/or high-fat foods and beverages [17]. In general, SNAP cannot be used to purchase prepared meals; however, Los Angeles County makes an exception for homeless individuals, the elderly, and the disabled, as well as their spouses [15], and the list of participating restaurants is dominated by fast food establishments [18].

All analyses were carried out with SAS v9.2 and sample weights were used to account for the complexity of the survey design [14]. We used descriptive statistics to characterize the sample and then estimated the unadjusted associations between obesity, SNAP participation, and density of unhealthy food outlets. To test for effect modification, we first mean-centered the modifier (density of unhealthy food outlets – mean density of unhealthy food outlets) to reduce multicollinearity between the interaction term (see next step) and the components of the interaction term and for ease in interpretation. After centering, we calculated the product interaction between SNAP participation and the mean-centered modifier. Given that the density of unhealthy food outlets is a census tract- level variable and individuals are nested within census tracts, we conducted a multilevel logistic regression analysis. Specifically, we created two regression models: 1) a base model, with obesity as our dependent variable, SNAP participation as our independent variable, and density of unhealthy food outlets, age, gender, race/ethnicity (non-Hispanic (NH) white, NH black, Hispanic, or other), marital status (married, living with a partner, or single), working status (currently employed yes/no), having a mental health problem (yes/no), and neighborhood poverty category (very poor, poor, non-poor) as covariates; and 2) an effect modification model, adding the product interaction term to the base model. These covariates were chosen because they were found to predict SNAP participation in a previous study with the same population [16] and/or because they were associated with obesity in the current analysis. A statistically significant interaction term would indicate that density of unhealthy food outlets is a modifier. If the interaction is not significant, the base model is used for parsimony.

## Results

Table 1 shows the distribution of selected sample characteristics by SNAP participation. In general, SNAP participants were younger, more likely to be African American, and less likely to be married, to have a high education, and to report an “other” ethnicity when compared to their eligible non-participants counterparts. When compared to eligible non-participants, SNAP participants had double the prevalence of obesity (30% vs. 16%) and were exposed to a higher neighborhood density of unhealthy food outlets (6.5 vs. 5.2 unhealthy food outlets per square mile).

**Table 1:** Selected characteristics of the sample of SNAP participants and eligible non-participants (*n* = 1176)

	SNAP participants (*n* = 412) %	SNAP eligible, non-participants (*n* = 764) %	*p*-value^1^
Age, years – mean (SE)	35.9 (1.1)	45.4 (1.3)	<.001
Gender (% female)	62.0	55.2	0.235
Race/ethnicity			<.001
Non-Hispanic white	34.1	32.3	Ref.
Non-Hispanic black	22.7	9.3	<.001
Hispanic	41.5	46.2	0.387
Other	1.9	12.2	<.001
Marital status			<.001
Married	35.2	48.1	Ref.
Living with partner (not married)	20.7	4.9	<.001
Single	44.0	46.9	0.009
Educational attainment			0.014
Less than high school	46.4	33.6	
High school or more	53.6	66.4	
Currently working (% yes)	35.0	48.6	0.013
Mental health problem (% yes)	19.9	6.4	<.001
Obesity (% yes)	30.4	16.5	0.004
Neighborhood poverty category			0.011
Not poor	45.3	52.7	Ref.
Poor	32.3	35.0	0.112
Very poor	22.5	12.3	<.001
Density of unhealthy food outlets (count per square mile) – mean (SE)	6.5 (0.5)	5.2 (0.3)	0.034

*SNAP*, Supplemental Nutrition Assistance Program

^1^ *p*-values obtained from weighted bivariate logistic regressions

Table 2 shows the results of the multilevel model. Compared to eligible non-participants, SNAP participants had double the odds of obesity, after adjusting for age, gender, race/ethnicity, marital status, working status, mental health, neighborhood poverty category, and density of unhealthy food outlets. Neighborhood density of unhealthy food outlets was not a significant predictor of obesity and it did not modify the relationship between obesity and SNAP participation since the interaction between SNAP participation and density of unhealthy food outlets was not significant (Table 2, second column). Restricting the sample to women only did not change the results (data not shown).

**Table 2:** Results of the multilevel effect-modification analyses between obesity,^a^ SNAP participation, and neighborhood unhealthy food outlets (*n* = 1,041)^b^

	Base model		Effect modification model	
	OR	95%CI	OR	95%CI
SNAP participation (ref = eligible non-participants)	2.02	1.44-2.83	2.09	1.48-2.95
Age (years)	1.02	1.01-1.03	1.02	1.01-1.03
Gender (ref = female)	0.45	0.30-0.67	0.45	0.30-0.67
Race/ethnicity (ref = Non-Hispanic white)				
Non-Hispanic black	2.79	1.45-5.38	2.76	1.43-5.32
Hispanic	2.15	1.30-3.56	2.12	1.28-3.51
Other	1.46	0.64-3.35	1.42	0.62-3.26
Marital status (ref = married)				
Cohabitating, not married	1.12	0.70-1.81	1.11	0.69-1.79
Single	0.94	0.67-1.32	0.93	0.66-1.31
Working status (ref = yes)	0.96	0.70-1.32	0.95	0.69-1.32
Mental health (ref = not having a mental health problem)	1.53	0.93-2.50	1.54	0.94-2.52
Neighborhood poverty category (ref = not poor)				
Poor	1.18	0.78-1.80	1.16	0.76-1.77
Very poor	1.02	0.67-1.57	1.01	0.66-1.55
Density unhealthy food outlets	1.01	0.98-1.03	1.00	0.97-1.03
SNAP participation* Density unhealthy food outlets			1.02	0.99-1.05

*SNAP* Supplemental Nutrition Assistance Program, *OR* Odds Ratio, *CI* Confidence Intervals

^a^Obesity defined as having a Body Mass Index ≥30 kg/m^2^

^b^Analytical sample includes SNAP participants and eligible non-participants only

## Discussion

We had hypothesized that being exposed to higher neighborhood density of unhealthy food establishments may lead to a stronger relationship between SNAP participation and obesity since respondents would have a greater chance of using their SNAP benefits in these outlets. Our findings did not support this hypothesis. Contrarily, a recent study has found that the regional density of SNAP-authorized stores is positively associated with obesity in metropolitan areas in the U.S. [19]. We did not have information about SNAP certification of the food outlets included in our analysis, however, and the presence of these outlets in the neighborhood is not equivalent to usage by SNAP beneficiaries. Moreover, we did not have the actual addresses of respondents and, therefore, were not able to estimate the distance of respondents’ homes to these food establishments. SNAP participants are likely to go outside their neighborhoods for SNAP purchases, especially if local food outlets do not accept SNAP benefits. Previous research shows that SNAP participants often carpool to farther but cheaper stores and sometimes visit multiple stores throughout the month to get better deals [20]. However, a study based on L.A. FANS data report that 34% of respondents shop for groceries at stores within a 15-min walk of their home and over 50% do their grocery shopping either in their own census tract or the neighboring one [21].

Han et al. [9] found that a large number of supermarkets and grocery stores in one’s zip code reduced the strength of the association between SNAP participation and BMI in a US representative sample. We used food environment indicators in the respondents’ census tract, which is a smaller geography than zip codes and therefore may better reflect neighborhood availability. In addition, we looked at a different dimension of the food environment than Han et al. [9], focusing on the density of unhealthy food outlets instead of on supermarkets and grocery stores. Even though the majority of SNAP-participating households report using supermarkets as their main type of food store [22], most also report redeeming SNAP benefits in other stores [23, 24], with 42% using convenience stores [24]. Moreover, Rigby et al. [7] found that the majority of SNAP-authorized stores in low-income and black and mixed-race neighborhoods in Leon County, Florida were not supermarkets or grocery stores but convenience and other stores, accounting for 69–75% of the SNAP-authorized stores available. Similarly, Shannon [8] found that in low-income, minority neighborhoods in Minnesota, 46% of SNAP redemptions take place in convenience stores. In addition, Laska et al. [25] found that SNAP-authorized small- to mid-size retailers in Minneapolis- St. Paul, Minnesota, did not stock a variety of healthy foods, particularly fresh foods and DeWeese et al. [26] found that SNAP-authorized corner stores in New Brunswick, New Jersey stock less healthy foods compared to non-SNAP vendors. Even though these geographical areas are not directly comparable to Los Angeles, we anticipate SNAP participants living in low-income and minority neighborhoods to have similar food accessibility issues as well as similar redemption and food purchasing patterns across the US. Research shows that the price of both healthy [9] and unhealthy foods [27] may modify the association between SNAP participation and obesity. Zhang et al. [27] found that higher prices of unhealthy foods (e.g. fast food, sodas) attenuates the association between SNAP participation and obesity (since SNAP participants would be less likely to purchase these expensive foods), whereas Han et al. [9] found that lowering the price of fruits and vegetables would have the same attenuating effect. Food price, store location, and transportation options are all likely to interact in SNAP participants’ decisions of where to make food purchases. Interestingly, a recent study found that proximity to food retailers did not modify the association between receiving a fruit and vegetable incentive and the purchase of such foods among SNAP participants in Hampden County, Massachusetts [28], implying that price incentives would benefit SNAP participants regardless of stores location. Los Angeles is heavily sprawled, however, with store location and availability of transportation likely playing a larger role in Los Angeles compared to other places.

One of the strengths of this paper is the extensive amount of income-related data available in L.A. FANS, which allowed us to identify a more accurate eligible non-participant group than in previous research linking SNAP participation and obesity, the vast majority of which classifies individuals as SNAP-eligible following simplistic income cut-off points (<130–185% federal poverty line) (e.g. [4, 29]). Additionally, predictors of SNAP participation in this population were identified in a previous study [16], so we were able to include these predictors as covariates in our analyses and reduce the impact of self-selection into SNAP in our results. Moreover, having access to yearly food environment data through NETS allowed us to get the counts of food establishments in the census tracts in the specific years where L.A.FANS respondents lived at the time of the survey. As for the limitations, the cross-sectional nature of this study makes it difficult to ascertain the temporality of the relationship between SNAP participation and obesity. Both BMI and SNAP participation are self-reported and subject to bias; however, the self-report of weights and heights by L.A. FANS respondents has been found to be valid [30]. Moreover, the data used in this study is relatively old (2000–2002), with data available at the census tract-level only. We cannot discard the possibility of different results if we had access to more current data, especially since some improvements in the food environment have taken place in Los Angeles since 2000–2002 [31], and/or data on smaller geographies. Furthermore, we did not account for population density, which may have confounded our results. Finally, the SNAP participation – obesity relationship has been found most consistently among women [2]. Given the small number of men in our sample (*n* = 278), we were unable to stratify our analysis by gender. However, our results remain the same if the sample is restricted to women only (data not shown).

## Conclusions

In sum, we found an association between SNAP participation and obesity as many others before us, highlighting the importance of promoting and facilitating healthy eating habits among SNAP participants. We also found that the neighborhood density of unhealthy food outlets was not a modifier in the SNAP participation – obesity relationship. More research is needed with different measures of the food environment (including availability, quality, and price) and additional measures of the physical environment where SNAP participants live (i.e. traffic and access to parks) to confirm these findings. Future studies looking at the food environments of SNAP participants should combine measures of exposure to neighborhood food outlets (quantity) with measures of quality of foods sold in these outlets by using existing validated tools such as those developed by the Nutrition Environment Measures Study (NEMS) [32, 33] or by measuring shelf space allocated to healthy vs. unhealthy foods. Future studies should keep investigating the mechanisms linking SNAP participation and obesity as they remain unclear. Longitudinal studies are needed to account for the temporality of the associations.

## Abbreviations

BMI: Body Mass Index
L.A. FANS: Los Angeles Family and Neighborhood Survey
NETS: National Establishment Time-Series database
SIC: Standard Industry Classification code
SNAP: Supplemental Nutrition Assistance Program
